# Prevalence of Neurological Symptoms and Imaging Findings in Von Hippel–Lindau Patients Referred to Rasool Akram Hospital, 2018–2021

**DOI:** 10.15586/jkcvhl.v11i2.313

**Published:** 2024-05-17

**Authors:** Seyed Hamidreza Javadi, Seyed Amir Hassan Habibi, Ahad Sedaghat, Masood Naseripour, Mohammad Yousefi

**Affiliations:** 1 Department of Neurology, School of Medicine, Iran University of Medical Sciences, Tehran, Iran;; 2 Eye Research Center, Rassoul Akram Hospital, Iran University of Medical Sciences, Tehran, Iran;; 3 Department of Medicine, School of Medicine, Semnan University of Medical Sciences, Semnan, Iran;; 4 Skull Base Research Center, The Five Senses Health Institute, School of Medicine, Iran University of Medical Sciences, Tehran, Iran

**Keywords:** Cerebellar tumors, Imaging findings, Neurological examination, Retinallesions, Von Hippel-Lindau

## Abstract

This study aimed to assess the prevalence of neurological symptoms and related imaging findings in patients with von Hippel-Lindau (VHL) at Rasool Akram Hospital from September 2018 to September 2021. This analytical observational study examined eligible patients over the period from September 2018 to September 2021. We collected demographic information (age, gender) along with imaging findings and results of neurological and eye examinations. Comparison between qualitative variables was also done using the Chi-square test or Fisher’s exact test. Also, an independent *t*-test was used to compare quantitative variables between the two groups. SPSS version 22 software was used for statistical analysis of data. A significant level was considered less than or equal to 0.05. Of the 54 examined patients (48.1% were male and 51.9% were female) with an average age of 36.42 ± 13.37 years. A significant majority (87.0%) reported a positive family history of the disease. The most common type of disease was Type 1 observed in 94.4% of cases and Type 2A was the next most frequent (3.7%). The most common pattern of retinal pathological lesions seen in the examination was related to bilateral lesions (79.6%). The most common pathological finding was related to the presence of a mass in cerebellar magnetic resonance imaging (48.1%). Considering the findings of the present study, which highlight a significant frequency of bilateral retinal lesions as well as masses in the central nervous system and endocrine system, it is evident that patients require careful follow-up and various interventions after being diagnosed with the disease. This approach is essential to manage and potentially mitigate the complications associated with these conditions.

## Introduction

Von Hippel-Lindau (VHL) syndrome is a genetically inherited disease (autosomal dominant) that leads to the development of benign and sometimes malignant tumors in several organs. Benign lesions in this syndrome are characterized by newly formed blood vessels (hemangioblastoma). These lesions can lead to serious, potentially life-threatening complications, particularly when they affect critical tissues like the retina, spinal cord, brain, and inner ear. The growth of these vessels usually follows an unusual system that does not follow a specific pattern and many of them are irregular (1). Most common tumors in patients with VHL include central nervous system tumors and retinal hemangioblastomas, clear cell renal carcinomas, pheochromocytomas, pancreatic neuroendocrine tumors, pancreatic cysts, intralymphatic sac tumors, and epididymal papillary cystadenomas. It is a mutation in the VHL tumor suppressor gene, which is located on chromosome 3p 25.3. (2). VHL disease occurs in approximately 1 in every 36,000 live births. These patients suffer from benign and malignant lesions during their lifetime, which leads to a reduction in the life expectancy of these patients to 40–52 years. According to studies, there have been about 200,000 confirmed cases of this disease in the world. Notably, 20% of these cases are de novo, meaning they occur for the first time in the family (1). During the studies, the most important causes of morbidity and mortality in these patients were hemangioblastoma and endolymphatic sac tumors in many parts of the body, especially the central nervous system (3). So far, studies in different countries of the world in terms of epidemiology, types of conflicts of this disease, and its clinical characteristics have been conducted. However, no coherent study has yet been undertaken in Iran. Therefore, this study aims to investigate the neurological and ophthalmological characteristics as well as the features of magnetic resonance imaging (MRI), to provide a clearer understanding of the disease’s impact within this population.

## Materials and Methods

This observational-analytical study was conducted at Rasool Hospital authorized by the ethics committee of the Iran University of Medical Sciences with the ethical number IR.IUMS.FMD.REC.1401.301. Following the acquisition of necessary permissions, the information of all patients with VHL’s disease who attended the neurology clinic of this hospital for neurological examinations was collected and recorded.

### Case selection

The definitive diagnosis criteria for VHL patients based on Hes et al.’s article are as follows (4):

If there is a positive family history of VHL disease, diagnosis can be confirmed by the presence of any one of the following:

- Retinal hemangioblastoma
- Brain hemangioblastoma
- Visceral lesions

In the absence of a family history of the disease, diagnosis requires the presence of any of the following:

- Two or more hemangioblastomas in the retina
- Two or more hemangioblastomas in the brain
- Both a retinal and a brain hemangioblastoma, accompanied by a visceral lesion

It should be mentioned that within the diagnostic criteria, the term “visceral lesions” refers to the observation of any of the following conditions: kidney cyst, kidney carcinoma, presence of pheochromocytoma, pancreatic cyst, islet cell tumor, epididymal cystadenoma, and endolymphatic sac tumor. In addition, the Logarithm of the Minimum Angle of Resolution (log-mar) scale was used to assess the visual acuity. For cases where the patients were examined only with the Snellen chart, logMAR scores were subsequently calculated for these patients using the corresponding conversion table (Table 1).

**Table 1:** Conversions between LogMAR and Snellen Visual Acuity Scores.

logMAR	Snellen equivalent
1.0	6/60
0.9	6/48
0.8	6/38
0.7	6/30
0.6	6/24
0.5	6/19
0.4	6/15
0.3	6/12
0.2	9.5/6
0.1	7.5/6
0	6/6
−0.1	5/6
−0.2	4/6
−0.3	3/6

### Eligibility Criteria

The inclusion criterion in this study was:

- All patients who were referred to the neurology clinic of Rasool Akram Hospital for routine examinations of VHL disease and visited in the period of September 2018 to September 2021.

The exclusion criterion was:

- Patients who did not visit the eye clinic for eye examinations.

### Data extraction

A preprepared checklist was used in this study. The information of each patient was entered into these checklists separately, and after collecting the information of all patients, the data in the checklist were then entered into the SPSS software. For the acquisition of imaging data, all subjects underwent annual MRI of the brain and entire spine, conducted both with and without contrast enhancement. The most recent imaging studies available were analyzed for this investigation. In addition, all participants underwent abdominopelvic sonography. Based on the sonographic findings, further diagnostic procedures, such as abdominopelvic computed tomography (CT) with contrast, were performed, particularly in cases where symptoms suggestive of pheochromocytoma were present.

### Statistical analysis

The results for quantitative variables were presented as mean and standard deviation, while qualitative variables were expressed using frequency percentages. Comparison between qualitative variables was conducted using the Chi-square test or Fisher’s exact test. An independent *t*-test was used to compare quantitative variables between the two groups. SPSS version 22 software was used for statistical analysis of data and a significance level of 0.05 or less was considered statistically significant

## Results

### Descriptive analysis

Of the 54 examined patients, 48.1% were male and 51.9% were female with an average age of 36.42 ± 13.37 years; most patients (87.0%) had a positive family history. Two of the examined patients (3.7%) had died. The most common type of disease observed was Type 1 (94.4%) and Type 2A was the next most frequent type of disease (3.7%). The most common pattern of retinal pathological lesions seen in the examination was related to bilateral lesions (79.6%). Only 13% of patients did not have any pathological lesions in retinal examination.

The average duration of the disease (from the initial diagnosis to the time of the study) was equal to 12 ± 9.11 years. During the examination of the visual acuity of the left and right eyes, it was found that the average visual acuity was 0.41 ± 0.45 and 0.42 ± 0.56, respectively.

### Distribution of abnormal imaging lesions

The distribution of abnormal imaging lesions in the studied patients is shown in Table 2; based on the information provided, the most common pathological finding is related to the presence of a mass in the cerebellar MRI (48.1%) and the rarest pathological finding is also related to the frequency of adrenal lesions, which was 5.6%.

**Table 2:** The presence of abnormal lesions in imaging.

Type of abnormal lesion	Number	Frequency (%)
Adrenal lesions	3	5.6
Kidney lesions	15	27.8
Pancreatic lesions	8	14.8
The presence of a mass in MRI of the cerebellum	26	48.1
The presence of a mass in brainstem MRI	8	14.8
The presence of a mass in MRI of the spinal cord	9	16.7

### Distribution of abnormal neurological examination

The state of distribution of abnormal neurological examination findings in the examined patients is shown in Table 3; based on the information provided, the most common abnormal neurological examination findings are related to cerebellar examinations (50.0%) and the rarest findings are related to sensory tests . The abnormality of the neurological examination was also related to sensory examinations with a frequency of 18.5%.

**Table 3:** The presence of abnormal lesions in neurological examinations.

Neurological examination		Number	Frequency (%)
Sensory tests	Normal	44	81.5
	Abnormal	10	18.5
Cerebellar examinations	Normal	27	50
	Abnormal	27	50
Pyramidal examinations	Normal	42	77.8
	Abnormal	12	22.2

### Pattern of clinical findings of patients

The results of chi-square analysis to examine the distribution pattern of clinical findings of patients, including family history, subtype of disease, and retinal lesions by gender, are shown in Table 4. There were no significant differences in the distribution of family history (P=0.61) and disease subgroup (P=0.22) by gender. While the frequency of patients without retinal pathological lesions was significantly higher in males and the frequency of bilateral retinal pathological findings was significantly higher in females (P<0.001).

**Table 4:** Qualitative clinical characteristics of patients by gender.

Clinical characteristic		Male (%)	Female (%)	P-value
Family History	Positive (n=47)	46.8	53.2	0.61
	Negative (n=7)	57.2	42.8	
Type of VHL	Type I (n=51)	51	49	0.22
	Type 2A (n=2)	0	100	
	Type 2B (n=1)	0	100	
Retinal lesion	No lesion (n=7)	85.7	14.3	0.00
	Left eye (n=4)	100	0	
	Right eye (n=0)	0	0	
	Bilateral (n=43)	37.2	62.8	

### Gender-based analysis

Based on the chi-square analysis to examine the difference between frequency of neurological examination findings and imaging findings according to gender, in most cases, the frequency of examination and imaging findings did not show a significant difference between male and female(P<0.05). Also, the frequency of any of the abnormal findings found during the neurological examinations did not have a significant difference between male and female patients.

The results of the *t*-test for comparing quantitative data such as age, duration of disease, and visual acuity by gender showed the relative similarity of the data by gender (the aforementioned data were not significantly different by gender).

## Discussion

In the present study, the patients with VHL’s disease referred to the nerve referral center were examined. Considering the very low prevalence of this disease, understanding the demographic patterns and clinical characteristics of the patients in the region is of significant importance.

For disease management in Iran, the initial approach includes a neurological examination along with MRI of the brain and spine. Patients are evaluated for pheochromocytoma through testing of serum metanephrine, normetanephrine, and 24-hour urinary vanillylmandelic acid (VMA) levels. On the first visit, abdominopelvic ultrasonography is also performed. If a malignant lesion is suspected, an abdominopelvic CT scan, both with and without contrast, is conducted. Additionally, abdominopelvic ultrasonography is performed annually in patients presenting with symptoms of kidney lesions.

For renal cystic hamartoma (RCH), follow-up visits are scheduled at 1, 3, and 6 months posttreatment. If no anomalies are detected during these follow-ups, the patient undergoes an ophthalmological examination every 6 months. Furthermore, it is crucial to evaluate all family members for RCH after a diagnosis is confirmed. According to the results obtained during the present study, the examined patients were relatively equally male and female, and more than two-thirds of them mentioned a family history of the same disease.

Previous VHL genetic study on this population demonstrated that among 17 families with RCH, 10 different types of VHL variants were identified. The most common variants that affected the structure of a domain of the VHL protein (pVHL) were missense mutations. Also, the majority of mutations were located on a domain in patients with central nervous system hemangioblastoma and RCH (5).

Most of the patients in this study have Type 1 VHL disease and present with bilateral retinal pathological lesions. During the investigations, it was determined that a predominant proportion of patients lacking observable retinal lesions during examination were male, while a majority of patients manifesting bilateral retinal lesions were also male. Eye complications in patients with VHL are divided into two main categories: angiomatous and nonangiomatous (6). Although retinal hemangioblastoma is one of the most common clinical findings of VHL disease, it is difficult to accurately estimate the prevalence of retinal lesions in these patients, because most of the studies that investigated these patients were case series studies. Due to the limited sample size, estimated prevalence will not be associated with such high accuracy. In their study, Singh et al. found retinal vascular hemangioma to be the most common and one of the earliest symptoms in patients with VHL. During the mentioned study, the prevalence of retinal vascular hemangioma was reported from 49 to 85% (7). In another study published by Varshney et al., retinal vascular hemangioma with a prevalence of 60% was reported as the second most common finding in patients with VHL, while the most common finding in the study was hemangioblastoma of the central nervous system (8). Despite the different prevalences of retinal lesions presented in various studies, there is still a consensus that the occurrence of retinal lesions is one of the first manifestations of this disease. Published data indicate that while retinal lesions often appear individually, up to a third of patients may exhibit multiple unilateral lesions, and up to half of the patients may have bilateral retinal lesions (7). However, information provided was not consistent with the findings of our study which showed that more than two-thirds of the patients examined in the present study had bilateral lesions. In a cross-sectional study published by Wong et al., 890 patients with VHL were examined, of which 335 had retinal involvement. Among patients with retinal lesions, 42% had unilateral lesions and 58% had bilateral lesions (9). According to the results of various studies, it seems that the distribution of retinal involvement in different populations shows a similar pattern but different frequencies, so in most studies, the frequency of monocular involvement is less and the frequency of binocular involvement is more. However, the percentage of frequency reported in the studies has been significantly different.

Masses of the central nervous system, primarily hemangioblastomas, are common in patients with VHL syndrome, with various studies reporting a prevalence of 60–68% for these lesions. Although these lesions are benign, they are one of the main causes of death and morbidity in patients with VHL due to their compressive effects.

In our study, cerebral space-occupying lesions were observed in about half of the patients, but it should be noted that the mortality rate in our studied patients was less than 5% of all patients. Based on the values reported in the study of Lonser et al. (10) and Chittiboina et al. (11), the prevalence of cerebellar lesions was 16–69%, brain stem 5–22%, and spinal cord 13–53%. The prevalence of space-occupying lesions reported in these studies is similar to the findings of our study and confirm the model presented in the present study. According to the available documents, tumors of the central nervous system show different growth patterns in patients with VHL syndrome. However, the highest tumor burden is related to relative germline mutations and male gender (8). Despite the fact that the distribution of tumors related to the cerebellum and spinal cord is not related to gender, tumors which were observed in the MRI examination in the brain stem area were significantly more common in males in this study. However, population bias should be considered for interpretation of the results of this study. Also, in the case of the growth of masses of the central nervous system, the possibility of neurological symptoms increases due to the compressive effect of these masses, the neurological manifestations that appear during clinical examinations should also be considered, which according to the findings of our study were the most common abnormal findings in neurological examinations related to cerebellar examinations.

Renal manifestations in patients with VHL mainly include benign renal cysts and RCC (1, 12). Multiple bilateral renal cysts have been observed in 50 to 70% of patients with VHL disease (11). In our study, imaging-detected renal lesions were observed in less than 30% of patients, which was similar to the prevalence reported in the study by Ashouri et al. for patients with VHL who also had renal cell carcinoma (RCC) at the same time (13). Of course, it should be noted that in the current study, the specific nature of kidney masses (whether cystic or malignant) was not accurately reported. This detail will be addressed in the strengths and weaknesses section of our analysis. According to the results of Cassol et al.’s study (14), pheochromocytoma has a prevalence of 20% in patients with von Wippel-Lindau which can appear bilaterally and unilaterally. The findings of the mentioned study were inconsistent with this study, in such a way that 5.6% of the patients had adrenal gland mass. It should be mentioned that due to the release of catecholamines, pheochromocytoma causes symptoms such as hypertension, tachycardia, and headache, and the diagnostic criteria for reporting the prevalence of pheochromocytoma in Cassol et al.’s study is different from our study. In our study, the frequency of suprarenal masses has been investigated and reported.

According to published studies, pancreatic lesions are found in 35–70% of patients with von VHL (10, 12, 14–16). In 12% of patients, pancreatic cysts are diagnosed as the only sign of VHL (11, 14). In our study, the frequency of pancreatic masses was less than in the mentioned studies. According to the studies, some pancreatic masses are diagnosed after autopsy (17), so it is possible that a larger fraction of the patients in our study also have pancreatic masses that are still undiagnosed.

## Conclusion

Considering the findings of the present study, which highlight a significant frequency of bilateral retinal lesions as well as masses in the central nervous system and endocrine system, it is evident that patients require careful follow-up and various interventions after being diagnosed with the disease. This approach is essential to manage and potentially mitigate the complications associated with these conditions.
